# Antiangiogenic therapy for primitive neuroectodermal tumor with thalidomide: A case report and review of literature

**DOI:** 10.1097/MD.0000000000009272

**Published:** 2017-12-22

**Authors:** Qing Li, Yong Liu, Yang Yu

**Affiliations:** Department of Oncology, Xuzhou Central Hospital Affiliated to Dongnan University, Xuzhou, Jiangsu, China.

**Keywords:** antiangiogenesis therapy, cancer, high throughput sequencing, PNETs, thalidomide

## Abstract

**Rationale:**

Peripheral primitive neuroectodermal tumor (PNET) is a kind of small round cell tumor derived from primitive neuroectodermal tumor.

**Patient concerns:**

PNET is a highly malignant tumor that is subordinated to Ewing sarcoma. It occurs predominantly in soft tissue and bone and rarely in the bronchi and lung. Traditional surgery, radiotherapy, and chemotherapy are used for the treatment of PNET, but are usually ineffective.

**Diagnoses:**

There was a rare case of a 17 year-old man diagnoses with primary pulmonary PNET.

**Interventions:**

The patient was treated by the remedy treatment with thalidomide after the poor effect of conventional radiotherapy and chemotherapy.

**Outcomes:**

The patient survived without disease progression for 15 months and was in stable condition.

**Lessons:**

Thalidomide provides a choice for maintenance therapy in PNET.

## 1 Introduction

Peripheral primitive neuroectodermal tumors (PNETs) are rare tumors occurring mostly in children and adolescents with a slight preference for males.^[1]^ PNET lacks specific clinical manifestations, and the main symptoms are pain and lumps at the location of the disease. PNETs are highly malignant tumors with rapid tumor progression. The treatment for PNETs includes surgical resection, chemotherapy, and radiotherapy. The commonly recommend chemotherapy regimens often includes several cycles of agents such as cyclophosphamide, vincristine, doxorubicin, etoposide, and ifosfamide.^[2,3]^ Several studies have reported poor long-term survivals in PNETs despite of multimodality treatment regimens.^[4,5]^ Although vascular targeting agents have been reported to produce some beneficial effect on Ewing sarcoma,^[6,7]^ the use of vascular targeting agents for the treatment of PNET has not been reported in the literature. Here, we reported a case of a 17-year-old PNET patient who was successfully treated with thalidomide, an antiangiogenic agent.

## 2 Case report

### 2.1 History

A 17 year-old man, who visited to Anhui Chest Hospital in August 2014, presented with cough with sputum, breathless, and fever. Computed tomography (CT) images showed lung cancer in the right lung with lower lobes atelectasis. Tracheoscopy indicated malignant small round cell tumor. The values of serum tumor markers such as NSE, CEA, SSC, and Cyfra 21–1 were not remarkable. The patients had no history of smoking and no family history of cancer.

### 2.2 Immunohistochemistry

**Figure 1:**
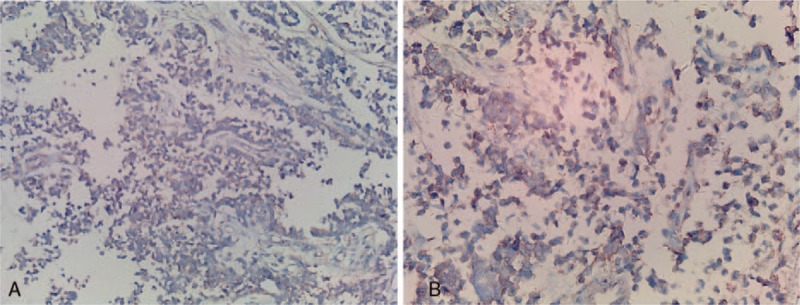
Immunohistochemical staining revealed that the tumor cells were positive for CD99 (Fig. 1A ×200), (Fig. 1B, ×400).

Immunohistochemical staining revealed that the tumor was positive for CD99 (Fig. 1A, B), Syn, CD56, Ki 67, Vim, and S-100, but negative for Cg-A, myoD1, CK, Des, myogenin, and SMA. CD99 plays an important role and differentiating diagnosis from other small-round cell malignant tumors. The diagnosis of Ewing sarcoma/primitive neuroectodermal tumors was made due to CD99 positive expression (Fig. 1A, B).

### 2.3 High throughput sequencing

Second-generation sequencing was used to detect the mutations of 16 tumor-related driver genes in peripheral blood and biopsies. The oncogenic mutation spectrum includes BRAF 54, EGFR 66, KIT 36, KRAS 34, PDGFRA 23, PIK3CA 29, CSF1R 7, ERBB2 16, FLT3 24, KDR 11, MET 9, NRAS 27, RET 13, SMO 5, SRC 1, and TP53. In these genes, 428 known-mutation regions were detected, and 5 mutations were present, including 4 mutations in uncommon mutation regions and 1 mutation in known mutation region (Table 1). All these mutations were synonymous mutations. No missense mutation and abnormal proteins were found.

**Table 1:**
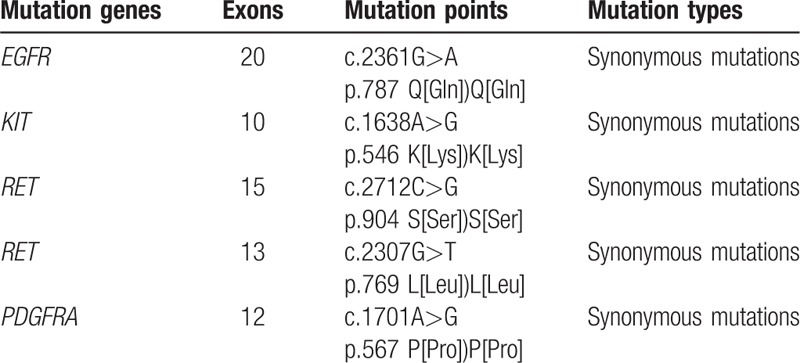
The mutation results of 5 mutation genes.

### 2.4 Treatment

The patient received chemotherapy and radiation therapy immediately after diagnosis. Although the initial treatment with VAC-IE (intensive vincristine + ifosfamide + etoposide + cyclophosphamide + doxorubicin) resulted in partial response, the treatment failed and the disease was progressive (Table 2). After 2nd-line chemotherapy of GP (gemcitabine + cisplatin) failed (Table 2), the patient was transferred from Shanghai Tumor Hospital to our hospital on September 3, 2015. However, TI (temozolomide + irinotecan) failed to improve the patient's condition, and the patient's health condition continued to deteriorate. The patient then received 3 cycle of chemotherapy TI (Table 3) plus thalidomide as salvage therapy. All the symptoms including pant, cough, and expectoration were relieved. During the follow-up period, he was given with monotherapy thalidomide (200 mg/day for 21 days) and remained in a good condition. Ethical approval was given by the medical Ethics Committee of Xuzhou Central Hospital, and the patient has signed the informed consent form.

**Table 2:**
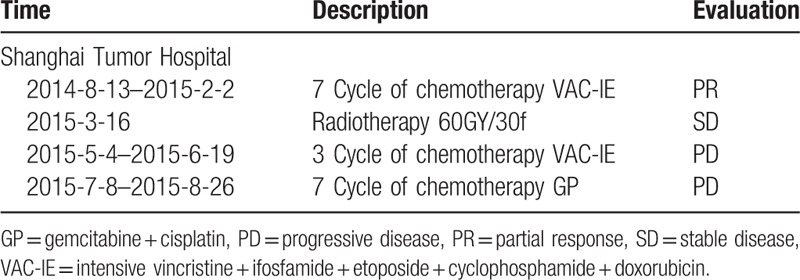
History of treatment (part 1).

**Table 3:**
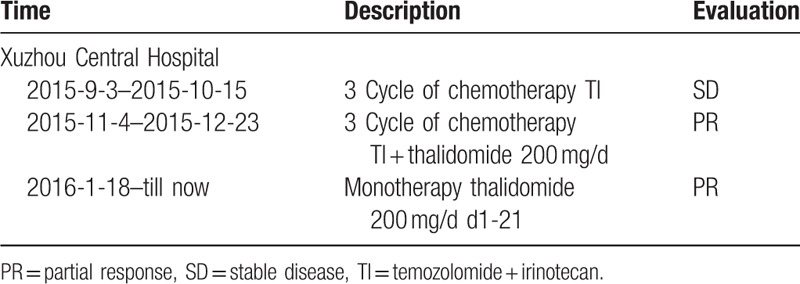
History of treatment (part 2).

### 2.5 Assessment

**Figure 2:**
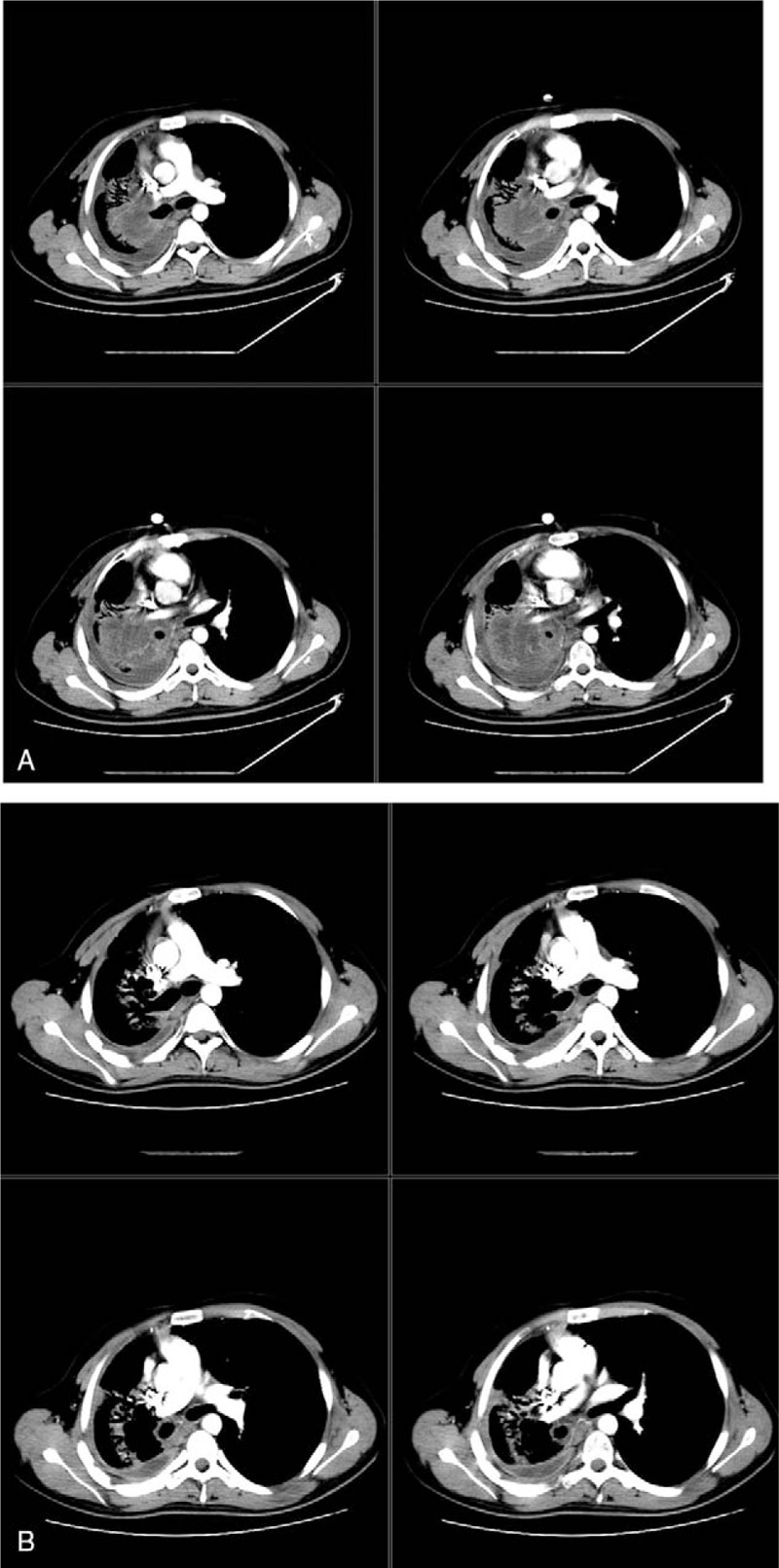
Thoracicoabdominal computed tomography (CT) scan found a soft tissue mass in the right lung. The mass had a maximum diameter of 88 mm × 82 mm. The mediastinum was shifted to the right side (A). The mass was eliminated dramatically (B).

The initial thoracicoabdominal CT scan showed a soft tissue mass with 88 mm × 82 mm in size in the right lung (Fig. 2A). The mediastinum was shifted to the right side, and mediastinal lymphadenopathy was found with pericardial effusion and pleural effusion. In contrast, recent CT results showed that the mass was decreased dramatically (Fig. 2B).

### 2.6 Follow-up

The patient now was alive with no sign of recurrence and went back to the normal life and social activities. He was followed up regularly at our outpatient clinic. He presented with no obvious symptoms such as cough, expectoration, and fever. He received maintenance dose of thalidomide (200 mg/day). Except mild hair loss, no obvious thalidomide-associated side effects such as rash, fatigue, thrombosis, and leukopenia occurred. The patient survived for 30 months till the last follow-up in March 2017.

## 3 Conclusion

In this case of a patient with PNET, oral thalidomide resulted in almost completely disappearance of the tumor, and was associated with mild adverse reaction.

## 4 Discussion

Primitive neuroectodermal tumors (PNET) belong to the Ewing sarcoma family of tumors, and preferentially occur in children and adolescents, with an age of younger than 35 years.^[1]^ PNETs most commonly originate from the longitudinal bone, such as the femur and humerus, and were also found in the pharynx, liver, and kidney.^[8–10]^ The tumors in the thoracopulmonary region were first reported as “malignant small cell tumors of the thoracopulmonary region in childhood” by Askin et al.^[11]^ Microscopically, the tumor consisted of uniform size of small round cell malignant tumor cells and Homer–Wrightrosettes was commonly present. PNETs exhibit a high sensitivity and specificity for positive immunohistochemical staining for CD99 antigen. Most studies have found that PNET has a CD99-immunopositive rate of >90%. CD99 is one of the most important immunohistochemical markers for the diagnosis of PNET.^[12]^ Dong et al^[13]^ summarized 20 PNET cases reported in the past 2 decades and found that there were 9 cases with the overall survival of 23.6 months (from 5 to 54 months), while there were still 9 cases alive with mean survival time over 17.2 months. These data suggest that the survival time of primary lung PNET was less than 2 years even after multiple treatments. VACA-IE is associated with improved 3-year disease-free and overall survival rates compared with the conventional chemotherapy regimen.^[14]^ It has been reported that the treatment effect was not significant different between local surgery and radiation therapy, and postoperative adjuvant radiation therapy would be beneficial for the preservation of organ function and the improvement of the clinical outcome.^[15]^ In this case, CT scan showed that the mass has uneven density with different degree of enhancement. Liquefied necrosis occurred in the center of the tumor, which is the sign of high invasiveness and rapid growth of tumors or blood vessel necrosis. Thalidomide was initially prescribed to treat cachexia. However, the tumor was inhibited by thalidomide. Folkman^[16]^ proposed the theory of “tumor growth and metastasis are dependent on angiogenesis.” Tumor angiogenesis plays an important role in the growth and metastasis of solid tumors,^[17]^ and antiangiogenesis therapy had proved to be a common and effective treatment for tumors. Several studies demonstrated that vasculogenesis was involved in the growth and development of Ewing sarcoma in mice, and vascular-targeted therapy might be the good alternatives for the treatment of Ewing sarcoma.^[6,7]^ Therefore, our finding that antiangiogenic agent thalidomide inhibited PNET growth may be due to its inhibition of high angiogensis of PENT.

Thalidomide, a derivative of glutamic acid, has been shown to be a potent antiangiogenic agent and immunomodulator. Several studies have demonstrated that thalidomide is effective for the treatment of multiple myeloma^[18]^ and cachexia.^[19]^ The phase II trials have showed that thalidomide in combination with radiotherapy and chemotherapy produces antitumor effects for many solid tumors, such as glioma,^[20]^ ovarian cancer,^[21]^ renal cell carcinoma,^[22]^ and prostate cancer.^[23]^ Thalidomide combined with chemotherapy has also been found to be effectively and safely used for the treatment of advanced colorectal cancer^[24]^ and liver cancer.^[25,26]^ Among these combinations, the efficiency of thalidomide-temozolomide treatment can significantly improve the efficacy for the treatment of melanoma. In a recent randomized phase II study,^[27]^ combined use of thalidomide and temozolomide enhanced the effectiveness and tolerance in patients with metastatic malignant melanoma.

Thalidomide has been found to produce antitumor effect via inducing tumor necrosis factor TNF-α degradation.^[28]^ Recent studies have found that thalidomide inhibits tumor angiogenesis by inhibiting the secretion of fibroblast growth factor.^[29]^ Thalidomide can inhibit tumor angiogenesis by inhibit vascular endothelial growth factor (VEGF) expression via targeting the VEGF promoter.^[30]^ In addition, thalidomide reduces the synthesis of integrin subunits, inhibits cyclooxygenase-2, and reduces microvessel density within the tumor. Additionally, thalidomide can activate CRBN-E3 ligase activity, leading to the ubiquitination and degradation of transcription factor IKZF1 and IKZF3, and eventually the disorder of B cells.^[31]^ Thalidomide can also enhance the immune system to kill tumor cells by activating T cell and reducing T cell inhibitory factor.^[32,33]^ In a retrospective analysis of advanced liver cancer, thalidomide monotherapy for more than 2 months prolonged the overall survival, and reduced alpha fatoprotein by more than 50%.^[34]^ Furthermore, thalidomide improves the quality of the terminal stage of cancer.^[35]^ However, of the benefit of thalidomide monotherapy for cancer has not been confirmed in large sample studies, and some studies have shown that thalidomide monotherapy has no significant effect in the treatment of metastatic head and neck cancer and breast cancer.^[36–38]^

Kaicker et al^[29]^ reported that thalidomide has antiangiogenic properties in neuroblastoma, and thalidomide was effectively used in patients with stage IV neuroblastoma. In a case of 5-year-old child with stage IV neuroblastoma, who had previously failed many chemotherapies, thalidomide produced a remarkable effect on metastatic neuroblastoma by reliving all symptoms. But unfortunately, the patient got recurrence quickly after withdrawal of thalidomide.^[39]^ In another case of a patient with refractory osteogenic sarcoma who refused chemotherapy, he survived more than 1 year by thalidomide alone.^[40]^ In a prospective, open-label, single-arm, multiinstitutional phase II study^[41]^ including 101 children with recurrent or progressive childhood cancer, multiagent treatment including thalidomide achieved complete remission in patients with Medulloblastoma/PNET. However, Pramanik et al^[42]^ reported that PNET patients did not benefit from antiangiogenic therapy including thalidomide and celecoxib, etoposide, and cyclophosphamide. These studies suggest that metronomic antiangiogenic chemotherapy may not benefit all kinds of pediatric solid tumors including PNET. Therefore, further studies with large sample size are required to investigate the benefit of antiangiogenic combination therapy.

## 5 Outlook

Thalidomide was invented in 1960s for the clinical treatment of decompensitivity and showed a significant teratogenic effect due to vascular inhibition during pregnancy. Now it has been used for the treatment of cancer. This case demonstrated that the clinical application effect of thalidomide is remarkable for the treatment of PNET. Clinically, thalidomide is effective in the treatment of refractory and hypervascula tumors. The use of thalidomide not only avoids the severe adverse reaction of cytotoxic drugs, but also avoids the occurrence of secondary tumor. Thalidomide provides a choice for maintenance therapy in child and adolescent with cancer. The long-term use of antiangiogenic drugs may constitute a new treatment for the maintenance of anticancer treatment. However, the long-term antitumor effects of thalidomide need to be studied in the future. Furthermore, further clinical studies are required to determine the dose, duration of treatment, and timing of treatment, and to assess efficacy, benefit, and adverse reactions as well as resistance of thalidomide.^[34]^
